# Supplementary material for: Effects of differing withdrawal times from ractopamine hydrochloride on residue concentrations of beef muscle, adipose tissue, rendered tallow, and large intestine
Source: PLoS One. 2020 Dec 2;15(12):e0242673. doi: 10.1371/journal.pone.0242673 (PMC7710041; doi:10.1371/journal.pone.0242673)
Supplement: S2 Table — (DOCX) [file pone.0242673.s002.docx]

**S2 Table.** Least squares means of total ractopamine (RAC) residue concentrations (ng/g) in muscle, adipose tissue, rendered tallow, and large intestine from steers fed one of five different treatments: (i) a negative control (fed no RAC and no feed-tallow; fed from verified clean feed trucks; Control-No Tallow); (ii) a control with feed-tallow (fed no RAC but received feed-tallow; Control-With Tallow); and cattle fed RAC (at approximately 250-300 mg/hd/day), but withdrawn from treatment at (iii) 2 days before harvest (2 day); (iv) 4 days before harvest (4 day); or (v) 7 days before harvest (7 day).

| Tissue/Matrix | Treatment | LSMean | 95% CI Low | 95% CI High | SE^1^ |
| --- | --- | --- | --- | --- | --- |
| Muscle | 2 day | 1.22^a^ | 1.06 | 1.38 | 0.08 |
|  | 4 day | 0.84^b^ | 0.68 | 1.00 |  |
|  | 7 day | 0.41^c^ | 0.25 | 0.57 |  |
|  | Control-With Tallow | < 0.12^d2^ |  |  |  |
|  | Control-No Tallow | < 0.12^d^ |  |  |  |
| Adipose tissue | 2 day | 0.50^a^ | 0.39 | 0.62 | 0.06 |
|  | 4 day | 0.32^b^ | 0.21 | 0.44 |  |
|  | 7 day | 0.18^b^ | 0.07 | 0.29 |  |
|  | Control-With Tallow | < 0.12^c3^ |  |  |  |
|  | Control-No Tallow | < 0.12^c3^ |  |  |  |
| Tallow | 2 day | 0.07^4^ | 0.06 | 0.09 | 0.01 |
|  | 4 day | 0.07^4^ | 0.05 | 0.09 |  |
|  | 7 day | 0.06^4^ | 0.04 | 0.08 |  |
|  | Control-With Tallow | 0.12^4^ | 0.03 | 0.07 |  |
|  | Control-No Tallow | 0.12^4^ | 0.04 | 0.07 |  |
| Large Intestine | 2 day | 8.45^a^ | 6.53 | 10.37 | 0.96 |
|  | 4 day | 5.71^b^ | 3.79 | 7.63 |  |
|  | 7 day | 4.80^b^ | 2.89 | 6.72 |  |
|  | Control-With Tallow | 0.88^c^ | 0.00 | 2.79 |  |
|  | Control-No Tallow | 0.76^c^ | 0.00 | 2.67 |  |

^1^SE denotes pooled standard error.

^2^ Denotes below the assay limit of detection for muscle (0.12 ng/g).

^3^Denotes below the assay limit of detection for adipose tissue (0.12 ng/g)

^4^ Values in red font are below the assay limit of quantitation for tallow (0.14 ng/g).

^abcd^Means without a common superscript within a column, by tissue/matrix, differ (*P* < 0.05).
