# Supplementary material for: Effects of differing withdrawal times from ractopamine hydrochloride on residue concentrations of beef muscle, adipose tissue, rendered tallow, and large intestine
Source: PLoS One. 2020 Dec 2;15(12):e0242673. doi: 10.1371/journal.pone.0242673 (PMC7710041; doi:10.1371/journal.pone.0242673)
Supplement: S3 Table — (DOCX) [file pone.0242673.s003.docx]

**S3 Table.** Parent and total ractopamine (RAC) residue concentrations (ng/g) in individual muscle samples from steers in each of the five experimental groups (i) a negative control (never fed RAC and never received feed-tallow during dosing; fed from verified clean feed trucks; “Control-No Tallow”); (ii) a control group that received feed-tallow (never receiving RAC, but received feed-tallow; “Control-With Tallow”); and cattle fed RAC plus feed-tallow, with withdrawal (iii) 2 days before harvest (“2 day”); (iv) 4 days before harvest (“4 day”); or (v) 7 days before harvest (“7 day”).

| Treatment | Parent RAC (ng/g) | Total RAC (ng/g) |
| --- | --- | --- |
| Control-No Tallow | < 0.12^*^ | < 0.12 |
|  | < 0.12 | < 0.12 |
|  | < 0.12 | < 0.12 |
|  | < 0.12 | < 0.12 |
|  | < 0.12 | < 0.12 |
|  | < 0.12 | < 0.12 |
|  | < 0.12 | < 0.12 |
|  | < 0.12 | < 0.12 |
|  | < 0.12 | < 0.12 |
|  | < 0.12 | < 0.12 |
|  | < 0.12 | < 0.12 |
|  | < 0.12 | < 0.12 |
|  | < 0.12 | < 0.12 |
|  | < 0.12 | < 0.12 |
|  | < 0.12 | < 0.12 |
| Control-With Tallow | < 0.12 | < 0.12 |
|  | < 0.12 | < 0.12 |
|  | < 0.12 | < 0.12 |
|  | < 0.12 | < 0.12 |
|  | < 0.12 | < 0.12 |
|  | < 0.12 | < 0.12 |
|  | < 0.12 | < 0.12 |
|  | < 0.12 | < 0.12 |
|  | < 0.12 | < 0.12 |
|  | < 0.12 | < 0.12 |
|  | < 0.12 | < 0.12 |
|  | < 0.12 | < 0.12 |
|  | < 0.12 | < 0.12 |
|  | < 0.12 | < 0.12 |
|  | < 0.12 | < 0.12 |
| 2 day | 0.51 | 1.01 |
|  | 1.09 | 2.46 |
|  | 0.89 | 1.36 |
|  | 0.62 | 1.06 |
|  | 0.58 | 1.14 |
|  | 0.62 | 0.93 |
|  | 1.03 | 1.07 |
|  | 0.71 | 0.96 |
|  | 1.10 | 1.57 |
|  | 0.96 | 1.51 |
|  | 0.74 | 0.78 |
|  | 0.39^†^ | 0.77 |
|  | 0.30 | 0.52 |
|  | 1.39 | 2.16 |
|  | 0.53 | 1.04 |
| 4 day | 0.55 | 0.68 |
|  | 0.53 | 0.71 |
|  | 0.42 | 0.48 |
|  | < 0.12 | < 0.12 |
|  | 0.26 | 0.45 |
|  | 0.61 | 0.78 |
|  | 0.80 | 1.03 |
|  | 0.76 | 1.08 |
|  | 0.69 | 0.99 |
|  | 0.32 | 0.60 |
|  | 0.53 | 0.66 |
|  | 0.27 | 1.05 |
|  | 1.23 | 1.50 |
|  | 0.36 | 0.78 |
|  | 1.51 | 1.72 |
| 7 day | 0.18 | 0.29 |
|  | 0.33 | 0.36 |
|  | 0.50 | 0.49 |
|  | 0.23 | 0.40 |
|  | 0.24 | 0.29 |
|  | 0.40 | 0.57 |
|  | 0.23 | 0.45 |
|  | 0.38 | 0.50 |
|  | 0.35 | 0.36 |
|  | 0.23 | 0.33 |
|  | 0.65 | 0.95 |
|  | 0.47 | 0.40 |
|  | 0.20 | 0.34 |
|  | < 0.12 | 0.17 |
|  | 0.42 | 0.26 |

^*^ < Denotes below the assay limit of detection (0.12 ng/g).

^†^ Values in red font are below the limit of quantification (0.41 ng/g).
