# Supplementary material for: Effects of differing withdrawal times from ractopamine hydrochloride on residue concentrations of beef muscle, adipose tissue, rendered tallow, and large intestine
Source: PLoS One. 2020 Dec 2;15(12):e0242673. doi: 10.1371/journal.pone.0242673 (PMC7710041; doi:10.1371/journal.pone.0242673)
Supplement: S4 Table — (DOCX) [file pone.0242673.s004.docx]

**S4 Table.** Parent and total ractopamine (RAC) concentrations (ng/g) in individual adipose tissue samples from steers in each of the five experimental groups (i) a negative control (never fed RAC and never received feed-tallow during dosing; fed from verified clean feed trucks; “Control-No Tallow”); (ii) a control group that received feed-tallow (never receiving RAC, but received feed-tallow; “Control-With Tallow”); and cattle fed RAC plus feed-tallow, with withdrawal (iii) 2 days before harvest (“2 day”); (iv) 4 days before harvest (“4 day”); or (v) 7 days before harvest (“7 day”).

| Treatment | Parent RAC (ng/g) | Total RAC (ng/g) |
| --- | --- | --- |
| Control-No Tallow | < 0.12^*^ | < 0.12 |
|  | < 0.12 | < 0.12 |
|  | < 0.12 | < 0.12 |
|  | < 0.12 | < 0.12 |
|  | < 0.12 | < 0.12 |
|  | < 0.12 | < 0.12 |
|  | < 0.12 | < 0.12 |
|  | < 0.12 | < 0.12 |
|  | < 0.12 | < 0.12 |
|  | < 0.12 | < 0.12 |
|  | < 0.12 | < 0.12 |
|  | < 0.12 | < 0.12 |
|  | < 0.12 | < 0.12 |
|  | < 0.12 | < 0.12 |
|  | < 0.12 | < 0.12 |
| Control-With Tallow | < 0.12 | < 0.12 |
|  | < 0.12 | < 0.12 |
|  | < 0.12 | < 0.12 |
|  | < 0.12 | < 0.12 |
|  | < 0.12 | < 0.12 |
|  | < 0.12 | < 0.12 |
|  | < 0.12 | < 0.12 |
|  | < 0.12 | < 0.12 |
|  | < 0.12 | < 0.12 |
|  | < 0.12 | < 0.12 |
|  | < 0.12 | < 0.12 |
|  | < 0.12 | < 0.12 |
|  | < 0.12 | < 0.12 |
|  | < 0.12 | < 0.12 |
|  | < 0.12 | < 0.12 |
| 2 day | < 0.12 | 0.44 |
|  | 0.17^†^ | 0.29 |
|  | 0.26 | 0.41 |
|  | < 0.12 | 0.24 |
|  | 0.48 | 1.33 |
|  | < 0.12 | 0.24 |
|  | 0.29 | 0.52 |
|  | 0.16 | 0.40 |
|  | 0.34 | 0.68 |
|  | 0.23 | 0.53 |
|  | < 0.12 | < 0.12 |
|  | 0.64 | 0.65 |
|  | < 0.12 | < 0.12 |
|  | 0.58 | 1.34 |
|  | 0.14 | 0.25 |
| 4 day | < 0.12 | 0.15 |
|  | 0.73 | 1.10 |
|  | < 0.12 | < 0.12 |
|  | < 0.12 | < 0.12 |
|  | < 0.12 | < 0.12 |
|  | < 0.12 | 0.28 |
|  | < 0.12 | < 0.12 |
|  | < 0.12 | 0.50 |
|  | < 0.12 | 0.31 |
|  | < 0.12 | 0.14 |
|  | < 0.12 | 0.14 |
|  | < 0.12 | 0.30 |
|  | 0.84 | 0.76 |
|  | < 0.12 | 0.19 |
|  | 0.46 | 0.51 |
| 7 day | < 0.12 | 0.13 |
|  | < 0.12 | < 0.12 |
|  | 0.24 | 0.38 |
|  | < 0.12 | < 0.12 |
|  | < 0.12 | < 0.12 |
|  | < 0.12 | 0.22 |
|  | < 0.12 | < 0.12 |
|  | 0.18 | 0.16 |
|  | 0.17 | < 0.12 |
|  | < 0.12 | < 0.12 |
|  | 0.37 | 0.60 |
|  | 0.21 | < 0.12 |
|  | < 0.12 | < 0.12 |
|  | < 0.12 | < 0.12 |
|  | < 0.12 | < 0.12 |

^*^ < Denotes below the assay limit of detection (0.12 ng/g).

^†^ Values in red font are below the limit of quantification (0.40 ng/g).
