# Supplementary material for: Effects of differing withdrawal times from ractopamine hydrochloride on residue concentrations of beef muscle, adipose tissue, rendered tallow, and large intestine
Source: PLoS One. 2020 Dec 2;15(12):e0242673. doi: 10.1371/journal.pone.0242673 (PMC7710041; doi:10.1371/journal.pone.0242673)
Supplement: S5 Table — (DOCX) [file pone.0242673.s005.docx]

**S5 Table.** Parent and total ractopamine (RAC) concentrations (ng/g) in individual rendered tallow samples (rendered in-laboratory from subcutaneous adipose tissue) from steers in each of the five experimental groups (i) a negative control (never fed RAC and never received feed-tallow during dosing; fed from verified clean feed trucks; “Control-No Tallow”); (ii) a control group that received feed-tallow (never receiving RAC, but received feed-tallow; “Control-With Tallow”); and cattle fed RAC plus feed-tallow, with withdrawal (iii) 2 days before harvest (“2 day”); (iv) 4 days before harvest (“4 day”); or (v) 7 days before harvest (“7 day”).

| Treatment | Parent RAC (ng/g) | Total RAC (ng/g) |
| --- | --- | --- |
| Control-No Tallow | 0.05^†^ | 0.07 |
|  | < 0.04^*^ | < 0.04 |
|  | < 0.04 | 0.08 |
|  | < 0.04 | 0.05 |
|  | < 0.04 | < 0.04 |
|  | 0.04 | 0.05 |
|  | < 0.04 | 0.12 |
|  | < 0.04 | < 0.04 |
|  | 0.08 | < 0.04 |
|  | < 0.04 | < 0.04 |
|  | < 0.04 | < 0.04 |
|  | 0.07 | < 0.04 |
|  | < 0.04 | < 0.04 |
|  | < 0.04 | < 0.04 |
|  | 0.06 | 0.04 |
| Control-With Tallow | < 0.04 | 0.05 |
|  | 0.06 | 0.05 |
|  | < 0.04 | < 0.04 |
|  | 0.12 | < 0.04 |
|  | 0.06 | < 0.04 |
|  | < 0.04 | < 0.04 |
|  | < 0.04 | < 0.04 |
|  | < 0.04 | < 0.04 |
|  | 0.05 | 0.07 |
|  | < 0.04 | < 0.04 |
|  | 0.07 | 0.11 |
|  | 0.06 | 0.07 |
|  | < 0.04 | < 0.04 |
|  | < 0.04 | < 0.04 |
|  | < 0.04 | 0.05 |
| 2 day | 0.07 | 0.07 |
|  | 0.05 | < 0.04 |
|  | 0.05 | < 0.04 |
|  | 0.09 | 0.12 |
|  | 0.10 | 0.05 |
|  | 0.05 | 0.05 |
|  | 0.15 | 0.16 |
|  | 0.09 | 0.05 |
|  | 0.07 | 0.05 |
|  | 0.05 | 0.06 |
|  | 0.06 | 0.06 |
|  | 0.07 | < 0.04 |
|  | 0.06 | 0.07 |
|  | 0.13 | 0.19 |
|  | < 0.04 | < 0.04 |
| 4 day | 0.07 | 0.08 |
|  | 0.05 | 0.11 |
|  | < 0.04 | 0.05 |
|  | < 0.04 | 0.11 |
|  | < 0.04 | < 0.04 |
|  | 0.07 | < 0.04 |
|  | < 0.04 | < 0.04 |
|  | 0.05 | < 0.04 |
|  | < 0.04 | < 0.04 |
|  | < 0.04 | < 0.04 |
|  | < 0.04 | 0.05 |
|  | 0.10 | 0.12 |
|  | 0.13 | 0.15 |
|  | < 0.04 | 0.05 |
|  | 0.06 | 0.08 |
| 7 day | < 0.04 | < 0.04 |
|  | < 0.04 | < 0.04 |
|  | < 0.04 | 0.09 |
|  | < 0.04 | 0.08 |
|  | < 0.04 | 0.05 |
|  | < 0.04 | 0.07 |
|  | < 0.04 | 0.06 |
|  | 0.06 | 0.07 |
|  | 0.09 | 0.04 |
|  | 0.06 | 0.06 |
|  | 0.09 | 0.09 |
|  | < 0.04 | < 0.04 |
|  | < 0.04 | < 0.04 |
|  | < 0.04 | < 0.04 |
|  | < 0.04 | 0.08 |

^*^ < Denotes below the assay limit of detection (0.04 ng/g).

^†^ Values in red font are below the limit of quantification (0.14 ng/g).
