# Supplementary material for: Effects of differing withdrawal times from ractopamine hydrochloride on residue concentrations of beef muscle, adipose tissue, rendered tallow, and large intestine
Source: PLoS One. 2020 Dec 2;15(12):e0242673. doi: 10.1371/journal.pone.0242673 (PMC7710041; doi:10.1371/journal.pone.0242673)
Supplement: S6 Table — (DOCX) [file pone.0242673.s006.docx]

**S6 Table.** Parent and total ractopamine (RAC) concentrations (ng/g) in individual large intestine samples from steers in each of the five experimental groups (i) a negative control (never fed RAC and never received feed-tallow during dosing; fed from verified clean feed trucks; “Control-No Tallow”); (ii) a control group that received feed-tallow (never receiving RAC, but received feed-tallow; “Control-With Tallow”); and cattle fed RAC plus feed-tallow, with withdrawal (iii) 2 days before harvest (“2 day”); (iv) 4 days before harvest (“4 day”); or (v) 7 days before harvest (“7 day”).

| Treatment | Parent RAC (ng/g) | Total RAC (ng/g) |
| --- | --- | --- |
| Control-No Tallow | < 0.32^*^ | < 0.32 |
|  | < 0.32 | < 0.32 |
|  | < 0.32 | < 0.32 |
|  | < 0.32 | < 0.32 |
|  | < 0.32 | < 0.32 |
|  | < 0.32 | 0.43^†^ |
|  | < 0.32 | < 0.32 |
|  | 2.27 | 2.57 |
|  | < 0.32 | < 0.32 |
|  | 0.92 | 0.81 |
|  | < 0.32 | < 0.32 |
|  | 2.95 | 4.00 |
|  | < 0.32 | < 0.32 |
|  | < 0.32 | < 0.32 |
|  | < 0.32 | < 0.32 |
| Control-With Tallow | 0.61 | 0.93 |
|  | < 0.32 | < 0.32 |
|  | < 0.32 | < 0.32 |
|  | < 0.32 | < 0.32 |
|  | 0.58 | 0.72 |
|  | < 0.32 | < 0.32 |
|  | 0.54 | 2.89 |
|  | 2.05 | 2.33 |
|  | < 0.32 | < 0.32 |
|  | < 0.32 | < 0.32 |
|  | < 0.32 | 0.60 |
|  | < 0.32 | < 0.32 |
|  | < 0.32 | < 0.32 |
|  | 2.38 | 2.83 |
|  | < 0.32 | < 0.32 |
| 2 day | 6.62 | 6.76 |
|  | 7.36 | 6.95 |
|  | 10.24 | 11.69 |
|  | 4.90 | 5.42 |
|  | 18.05 | 20.74 |
|  | 2.00 | 2.77 |
|  | 17.15 | 18.10 |
|  | 2.68 | 3.60 |
|  | 5.36 | 6.90 |
|  | 7.05 | 8.82 |
|  | 1.25 | 1.31 |
|  | 1.34 | 2.04 |
|  | < 0.32 | 1.14 |
|  | 15.22 | 16.13 |
|  | 12.06 | 14.37 |
| 4 day | 7.35 | 8.81 |
|  | 8.21 | 11.26 |
|  | 0.89 | 1.26 |
|  | < 0.32 | < 0.32 |
|  | 3.76 | 4.63 |
|  | 3.25 | 3.71 |
|  | 1.12 | 1.55 |
|  | 3.43 | 5.93 |
|  | 8.10 | 10.76 |
|  | < 0.32 | 1.29 |
|  | 3.47 | 4.50 |
|  | 9.86 | 11.04 |
|  | 13.84 | 12.15 |
|  | 6.04 | 6.58 |
|  | 1.41 | 1.85 |
| 7 day | 1.57 | 1.87 |
|  | 6.49 | 6.41 |
|  | 2.22 | 6.12 |
|  | 4.75 | 5.91 |
|  | 8.83 | 11.63 |
|  | < 0.32 | < 0.32 |
|  | 6.03 | 7.43 |
|  | 5.82 | 5.86 |
|  | 3.15 | 4.29 |
|  | 4.60 | 5.75 |
|  | 7.50 | 7.46 |
|  | 3.10 | 3.18 |
|  | 1.27 | 2.03 |
|  | < 0.32 | < 0.32 |
|  | 3.58 | 3.49 |

^*^ < Denotes below the assay limit of detection (0.32 ng/g).

^†^ Values in red font are below the limit of quantification (1.08 ng/g).
