# Supplementary material for: Effects of differing withdrawal times from ractopamine hydrochloride on residue concentrations of beef muscle, adipose tissue, rendered tallow, and large intestine
Source: PLoS One. 2020 Dec 2;15(12):e0242673. doi: 10.1371/journal.pone.0242673 (PMC7710041; doi:10.1371/journal.pone.0242673)
Supplement: Attachment — Submitted filename: PONE-D-20-27930 Review.pdf [file pone.0242673.s008.pdf]

PONE-D-20-27930, “Effects of Differing Withdrawal Times from Group Treatment with Ractopamine Hydrochloride on Residue Concentrations in Beef Muscle, Fat, Rendered Tallow, and Large Intestine” is a generally well-written, but vague article describing residues of ractopamine in beef cattle. The manuscript is publishable but requires major revisions.

General Comments.

1. The authors have provided no explanation for the basis of tissue selection. Lung and kidney, in addition to intestinal tissue, are major offal tissues destined for export. Please provide a rationale for the rather limited selection of offal tissues.
2. The practical uses of limit of detection and limit of quantitation poorly defined.
    a. The limit of detection for tallow is defined as 0.04 ng/g in Table 1 but is defined in Supplementary materials Tables 1 and 2 as 0.12 ppb.
    b. The authors report values below the limit of quantitation, but above the limit of detection (i.e., detectable residues) as quantifiable residues. In addition, the authors quantify and report residues below the LOD. For example, Figures 1a and 1b show 4 means below lines representing the LOD. Thus, it appears that the authors are quantifying and reporting what is, by *definition,* not quantifiable. In other words, the authors appear to be quantifying and reporting *background* as measurable residue.
    c. The authors have not provided how they calculate means from data sets having quantifiable, detectable, and non-detectable values. There are several methods for handling such data and I don’t really think it matters which method one uses, but the method should be disclosed to readers (in the materials and methods). For example, one could state something like “. . . within treatment and tissue, means were calculated using individual values of quantifiable residues, the nominal values returned for detectable residues, and the LOD value for non-detectable residues” (this is a fairly conservative method which will overestimate residues at the lower end of the scale). Again, there a lot of ways that values <LOQ and <LOD can be handled, but whatever method is used must be described in the materials and methods.
3. Experimental Design. Whatever the authors state about experimental unit (lines 94 to 98), “animal” is not the experimental unit. As the manuscript reads, the ractopamine-fed animals were fed in a single pen and then segregated by withdrawal period. Therefore “pen” is the experimental unit (unless there are individual feed intake data; if there are, then the dose per animal on a mg/kg bw basis could be calculated for the beginning, mid-point, and end of the feeding period . . . then one might be able to justify the experimental unit argument). Having said that, the argument then becomes is the amount of “within pen” variation representative of the amount of variation if the study had been spread among, say 3 pens or locations. We’ll never know. Suggest reporting the study as designed, without the argument that “animal” was the experimental unit. Again, within treatment, single pens of animals were fed, not individual cattle.
4. Conceptual Design. The inclusion of tallow as a potential source of ractopamine residues is curious as adipose tissue is a poor reservoir for ractopamine residues (ractopamine is not lipophilic; at physiologic pH it is a rapidly excreted cation; the glucuronide metabolites are zwitterions). The authors should provide some context in the discussion as to why tallow is a concern as a source of ractopamine residues. I’m not saying it couldn’t be of concern, but the choice of tallow should be addressed, especially as the data of Gressler et al. (Journal of Chromatography B, 1015–1016 [2016] 192–200) and Aroeira (Carolina N. Aroeira, Vivian Feddern, Vanessa Gressler, Luciano Molognoni, Heitor Daguer, Osmar A. Dalla Costa, Gustavo

J.M.M. de Lima & Carmen J. Contreras-Castillo [2019] Determination of ractopamine residue in tissues and urine from pig fed meat and bone meal, Food Additives & Contaminants: Part A, 36:3, 424-433, DOI: 10.1080/19440049.2019.1567942) demonstrate that meat and bone meal from ractopamine fed animals may contain ractopamine residues with potential for detectable 'carry over'.

5. The authors should consider using "adipose tissue" in place of "fat" when they are describing the tissue that was sampled and assayed. Sometimes "fat" (the tissue) is conflated (or at least equated with "fat" tallow. Fat is a component of adipose tissue, but not its sole component.
6. The authors have failed to provide adequate descriptions of the test animals. What was the proportion of steers and heifers? What was the duration of ractopamine exposure (i.e., the length of the feeding period?)? What were the initial and final weights of the experimental animals?
7. There is quite a body of extant literature on ractopamine residues in the context of feed contamination, trade, illegal use, and safety. The authors have accessed almost none of this literature, but some of it is relevant to their study. At a minimum the Brazilian studies of Gressler and Aroeira (mentioned above) should be included in the introduction and/or discussion.

Specific Items. (line, comment)

12	Here and throughout the manuscript, be cautious of conflating fat and adipose tissue; they are not synonymous.
35-36	Tallow values are reported as 0.05-0.08 ng/g, but these values are below the limit of detection reported in the supplementary data tables 1 and 2.
52	"with fewer inputs" sounds like "animal science jargon", but a broader audience will be accessing this journal. Please describe the economic, labor, energy, etc., "inputs" that are important for the reader to grasp.
57-58	Please provide a reference for the assertion that ractopamine is a pure beta-2 agonist. The authors may want to consider the papers by Mills et al (2003; Journal of Animal Science) which suggests that both beta-1 and beta-2 receptors are activated by ractopamine stereoisomers (i.e., commercial ractopamine is comprised of 4 stereoisomers in a roughly equimolar amounts).
77-70	The safety models used by the US FDA CVM assume that exposure to residues occurs daily *over a lifetime* (assumed to be 70 years). While this is sort of a technical detail, it has profound implications for setting tolerances (o,r in Europe, MRLs).
96-97	Suggest wording change; what exactly does "to reflect true application of the compound mean"? Perhaps "to reflect commercial application" is what the authors mean?
104-105	Fat is not a tissue type. Adipose tissue contains copious quantities of fat.
107-109	This sentence is out of place as it is essentially a result. It has nothing to do with how the study was conducted.
112-113	Suggest moving the IACUC approval to the beginning of the section where animal feeding is described. Chronologically, IACUC approval was obtained before the initiation of the experiment.
113-118	A random process is not described this section . . . far from it. Perhaps the sentence beginning at line 113 could be clarified by stating "Within treatment, the sequence of cattle loaded for shipment and for slaughter was random".
130	"proceeded down the line" reads like animal science jargon. Suggest using different wording.

131-133 A better description of the large intestine collection/processing is needed. No mention is made of whether the analyzed sample contained tissue and intestinal contents or just tissue. Because ractopamine is provided in the diet at very high levels (10 ppm dietary ractopamine = 10 µg/g = 10,000,000 ng/g) it's pretty important to know that intestinal contents were removed from the sampled tissue and how this process was accomplished. Is the measured 'tissue' ractopamine true residue incurred into intestinal tissue or unabsorbed gut lumen ractopamine of dietary origin?

133-134 Both subcutaneous (line 130) and KPH adipose tissue were collected. Why? KPH is not mentioned again in the manuscript. Which samples were used for adipose tissue analyses?

143 Is the 1 ± 0.5 g of tissue a typo? Read literally, the sample sizes analyzed ranged from 0.5 to 1.5 g . . . . this is a huge variance. If this is not a typo, then a better explanation of how differences in tissue mass were handled during the extraction method will be required. That is, was extraction solvent volume (and the amount of internal standard) adjusted for the sample weight?

167-168 It is implied that sample extract volume was dependent upon sample mass; if so, state explicitly. If not, then provide an explanation of how the 0.5 g samples were handled differently than 1.5 g samples. (see comment for line 143). "4 mL/g methanol" is ambiguous; read literally this means that solvent volume was added according to the grams of methanol added. Please reword.

180 Suggest rewording "analytical method" to read "instrumental method". The "analytical method" encompasses sample processing trough instrumental analysis.

186 Mobile phase B is not a buffer. Please reword.

212-214 There are several problems with the description of the calibration curve.

a) Literally read, a "Serial dilution" of both ractopamine and the internal standard means that the standard curve would cover 11 orders of magnitude for an 11 point curve (which is impossible for a curve covering 0.05 to 50 ng/mL). Please reword describing to the reader the use of stock solutions and intermediate standards in the preparation of the calibration curve.
b) As described, the amount of matrix at each point in the standard curve would differ unless matrix were used as the diluent. If matrix was used as the diluent in the preparation of the standard curve, please state that fact.
c) As stated, the concentration of the internal standard would not be consistent across the points of the standard curve (i.e., a serial dilution of a matrix matched stock solution of ractopamine and internal standard). Please reword.
d) "The standard curve range was optimized for each tissue to capture the appropriate concentration of the samples"; what does this sentence mean? It implies that standard curve preparation was tissue specific. If that is the case, then the description of the standard curve and the dynamic range of the standard curve is inadequate. Please clarify.
e) There is no mention of quality control criteria used to establish assay validity. Such criterial commonly include recovery of analyte fortified into control tissue and precision (scatter) of fortified samples; linearity of calibration curves; etc.

261 Suggest rewording, "67% had total RAC residue concentrations above the LOD" to "had detectable, but not quantifiable, RAC residues".

266-274 The steps used to remove intestinal contents from tissue were not described. Therefore, is not clear if the reported residues are residues from tissue or digesta. Please clarify.

281	The LOD provided is consistent with Table 1, but not with the LOD reported in the raw data (i.e. supplementary tables 1 and 2).
312-313	The FDA considers "fat" a traditionally edible tissue; it is not considered offal (Please see FDA CVM's Guidance for Industry No. 3; General Principles for Evaluating the Human Food Safety of New Animal Drugs Used In Food-Producing Animals). So, when a drug sponsor conducts residue studies, residues in adipose tissue must be measured. Adipose tissue is not an appropriate target tissue for ractopamine use because the amount of residue in adipose tissue is very low relative to liver (see the FOI summary for ractopamine).
319-321	The reader needs to understand the preparation of the intestinal samples . . . how were contents removed?
Figures 1 & 2	How are means in figures 1 and 2 calculated with respect to data sets that included values above and below the LOD? The method used by the authors to calculate means and error used in graphics should be clearly explained.
