# Supplementary material for: Effects of differing withdrawal times from ractopamine hydrochloride on residue concentrations of beef muscle, adipose tissue, rendered tallow, and large intestine
Source: PLoS One. 2020 Dec 2;15(12):e0242673. doi: 10.1371/journal.pone.0242673 (PMC7710041; doi:10.1371/journal.pone.0242673)
Supplement: Attachment — Submitted filename: Pone 27930.docx [file pone.0242673.s007.docx]

The experimental design and the data results shown in figures (1 A-D) and Tables (2-6) are very well presented.

Paper accepted in the current form.
